# Officials’ promotion expectation, corporate strategic deviance and corporate growth in China: The moderating effect of corporate ownership

**DOI:** 10.1371/journal.pone.0284872

**Published:** 2023-08-25

**Authors:** Yugang Li, Xiuyuan Fang

**Affiliations:** School of Business, East China University of Science and Technology, Shanghai, China; Universita degli Studi di Pisa, ITALY

## Abstract

Government (especially local government) plays an important role in China’s economic growth, the government is made up of officials, corporates are participants and the driving force of market economy, therefore, ignoring officials may not be able to directly explain the mechanism of corporate growth. This paper intends to discover how officials’ promotion expectation may be beneficial for corporates—directly and/or indirectly via corporate strategic deviance—in terms of corporate growth. We conduct an empirical analysis of Chinese listed companies to test these arguments, the results show that officials’ promotion expectation has a significantly positive impact on corporate growth; corporate strategic deviance has a mediating effect on the relationship between officials’ promotion expectation and corporate growth; compared with non-state-owned enterprises, corporate strategic deviance has less influence on state-owned enterprises’ growth. Our research generates a more comprehensive understanding of the political stakeholders-corporate growth relationship, provides direct evidence for the positive role of officials in corporate growth and expands the mediating research of corporate growth.

## 1. Introduction

Since the reform and opening in 1978, China’s economy has made great achievements, scholars interpreted the reasons for China’s economic growth from different perspectives. New institutional economists suppose that institution is important to economic development, and a country’s institution determines economic growth [1, 2]. However, according to the criteria of LLSV, China does not have advantages in property, capital market, legal and other aspects. More and more scholars believe that government (especially local government) plays an important role in China’s economic growth [3], government is made up of officials, we should not only consider "government" but also consider "officials”. Corporates are participants and the core driving force of market economy, ignoring officials may not be able to directly explain the mechanism of corporate growth. Although existing researches have made many efforts to understand corporate growth, there are still some limitations, for example, exiting researches often ignore the role of individual agent [4]. Therefore, there is a limit to explain regional economic development and corporate growth if we ignore officials who as the state agents.

Political sociology emphasizes the characteristics of bureaucracy (administrative rules and hierarchical structures) and its role in shaping officials’ behaviors [5], but it cannot explain why the same policy is implemented to different degrees in different places. Different from political sociology, political economists emphasize officials’ heterogeneous incentive and point out that officials may maximize their political career when making decisions [6], but political economy’s research neglects the constraint of the bureaucracy on officials. Based on political economy and political sociology, we believe that officials who as the government agents are influenced by bureaucracy [7] and their career stages [8]. Officials’ promotion expectation refers to officials’ psychological expectation for their promotion opportunities [9]. If officials’ promotion expectation differ, so will their priorities and their efforts to mobilize firms under their jurisdiction. Our research proposes that officials’ promotion expectation has a significantly positive impact on corporate growth; corporate strategic deviance has a mediating effect on the relationship between officials’ promotion expectation and corporate growth; the positive relationship between corporate strategic deviance and corporate growth will be weaker for state-owned enterprises.

This study aims to propose three important contributions in existing literature. First, we provide direct evidence for the positive role of officials in corporate growth. Existing research on the influencing factors of corporate growth mainly focuses on resources, strategy, and environment [10–17], our study provides a political explanation for corporate growth, it is helpful to understand the relationship between officials and corporates in emerging markets. Second, our research contributes to the discussion on the relations among officials’ promotion expectation, corporate strategic deviance and corporate growth. We analyze the mediating role of corporate strategic deviance between officials’ promotion expectation and corporate growth, it provides a more complete vision of the potential interrelations among them. Third, it contributes to corporate strategic deviance literature by examining the effects of corporate strategic deviance on corporate growth and the moderating effect of corporate attribute heterogeneity.

The institutional background of political centralization and economic decentralization in China makes it possible to study this issue: on the one hand, the central government decides local officials’ promotion [18], the central government has clear promotion requirements for officials and evaluates officials through three indicators: hard indicators (economic indicators), veto power and soft indicators (general indicators) [19]. Officials need to demonstrate their abilities and build their reputations by completing assessment indicators, such incentives prompt officials to allocate attention and resources strategically [20]. On the other hand, economic decentralization leads to the basic pattern of regional development dominated by local officials, officials play an important role in policy making, infrastructure construction, investment attraction and resource allocation [21]. Officials whose promotion expectation is low hope to a peaceful transition to retirement and opportunities that are appointed to positions of People’s Congress (CPC) or People’s Political Consultative Conference (CPPCC), while officials who have high promotion expectation are more interested in promotion. If officials’ promotion expectation differ, so will their priorities and their efforts to mobilize firms under their jurisdiction.

The article structure is as follows. Section 2 develops hypotheses on the effects of officials’ political incentive on corporate growth, the mediating effect of corporate strategic deviance on the relationship between officials’ promotion expectation and corporate growth, and the moderating effects of corporate ownership on the relationship between corporate strategic deviance and corporate growth. In section 3, the database, variables and models are reported. Section 4 presents empirical results. In section 5, the discussion is reported. Section 6 presents the theoretical implications and managerial implications. In section 7, limitations and conclusions are reported.

## 2. Hypothesis development

### 2.1 The direct effect of officials’ promotion expectation on corporate growth

Officials are encouraged to pursue multiple goals and are evaluated by hard indicators, veto power and soft indicators. Hard targets are economic indicators such as GDP growth rate, taxes, and foreign direct investment (FDI). Veto power is a political task, and the most important thing in China is to maintain social stability [22]. Soft targets refer to social welfare such as improving education and reducing environmental pollution. Compared with veto power and soft indicators, economic indicators are more likely to attract officials’ attentions due to it is related to officials’ promotion and is easy measured [23]. Excessive efforts on veto power and soft indicators are not directly related to officials’ promotion after meeting the minimum requirements. At the same time, officials cannot equally promote multiple goals’ realization due to their attentions and resources are limited [24], just as behavioral studies point out that it needs to balance when pursue multiple goals: the realization of one goal will sacrifice others [25]. Economic indicators are still the most important determinant of officials’ promotion [26], just as existing research found that the GDP growth rate of a province can positively predict provincial officials’ promotion to central government [27]. Officials whose promotion expectation is low hope to a peaceful transition to retirement and opportunities that are appointed to positions of People’s Congress (CPC) or People’s Political Consultative Conference (CPPCC), while officials who have high promotion expectation are more interested in promotion. If officials’ promotion expectation differ, so will their priorities and their efforts to mobilize firms under their jurisdiction. We believe that officials who have high promotion expectation have incentives to use resources and policies to promote corporate growth to obtain promotion.

Specifically, officials who have high promotion expectation could promote corporate growth in their jurisdictions through two ways: On the one hand, they could directly intervene in enterprises activities to stimulate economic growth, such as guiding enterprises investment by project, merger and acquisition. Just as Xu et al. found that officials’ promotion pressure leads to corporate growth through mergers and acquisitions [28]. On the other hand, they can promote corporate growth by providing scarce resources (such as land and finance) and policy support. First, officials can offer scarce resources (such as land) to corporates [29]; corporates can mortgage land to bank for loans, so they have enough capital in development. Second, officials can intervene in state bank to provide financial resources to companies. Although China’s financial market is constantly improving, state-owned banks still control most financial resources, officials could help corporates to obtain bank loans and reduce financing costs. Third, officials as policies designers and executors can influence corporate growth within their jurisdiction through policies. Officials who have high promotion expectation can selectively formulate and implement regional policies according to their promotion needs, such as using fiscal subsidies and tax rebates to promote corporate growth, to achieve rapid economic growth in the short term.

In summary, we believe that officials who have high promotion expectation could promote corporate growth. Specifically, they could intervene in enterprises activities, provide scarce resources and policy support to corporates in their jurisdictions. On this basis, we propose the following:

**Hypothesis 1**. Officials who have high promotion expectation have a positive effect on corporate growth.

### 2.2 The indirect effect of officials’ promotion expectation on corporate growth: The mediating role of corporate strategic deviance

We believe that firms in regions governed by officials who have high promotion expectation have higher strategic deviance. Specifically, officials who have high promotion expectation may give more opportunities to corporates whose strategic deviance is high but can promote regional economic development to obtain promotion due to they focus more attentions and resources on goals that could maximize their political career [30]. And officials’ supports for corporates whose strategy is not consistent with industry mainstream strategy benefit enterprises to obtain economic stakeholders’ supports, this is because government in China-as an institutional force-has a greater influence on corporates [31], economic stakeholders often judge whether enterprises are legitimate and whether to give more supports based on the interaction between enterprise and government [32]. Therefore, we believe that companies in regions governed by officials who have high promotion expectation are more likely have high strategic deviance.

We also believe that corporate strategic deviance has a positive effect on corporate growth. On the one hand, firms whose strategic deviance is high could attract more investors’ supports [33], just as resource-based view pointed out that uniqueness rather than imitation provides competitive advantage for enterprises to obtain resources [34]. Although firms with high strategic deviance face high costs which may increase the uncertainty of cash flow, existing studies found that cash flow uncertainty caused by strategic deviance can promote R&D investment and corporate value [35]. On the other hand, competitive pressure decreases with strategic deviance increase, and corporates have more opportunity to obtain leading position and high income. Firms whose strategic deviance is high could away from competition [36] and make themself in place where competition is insufficient. Based on these two reasons, we predict that corporate strategic deviance has a positive effect on corporate growth.

We study the indirect relation between officials’ promotion expectation and corporate growth, considering the mediating role of corporate strategic deviance. Therefore, we argue that corporate could use the resources and policy provided by officials through strategic deviance to promote corporate growth.

Officials who have high promotion expectation provides firms with an opportunity to deviate with industry mainstream strategy and benefit firms to obtain economic stakeholders’ supports. Officials who have high promotion expectation, then, is an effective method of obtaining scarce resources and policy support for corporate strategic deviance. Officials who have high promotion expectation, therefore, gives firms the opportunity, resources and policy support, advantages that can deliver higher corporate strategic deviance. For its part, corporate strategic deviance is also positively related to corporate growth. Firms whose strategic deviance is high display higher growth rates in so far as this deviance benefit them to attract more investors’ supports [33], to increase distance between them and competitors [37]. In line with this, firms whose strategic deviance is high are more likely to experience particularly rapid growth.

Therefore, we posit a chain of effects starting from officials’ promotion expectation to corporate strategic deviance and, ultimately, to corporate growth. That is, the role of officials’ promotion expectation is path dependent, corporate strategic deviance represents the intermediate channels that account for the effects of officials’ promotion expectation on corporate growth. Based on the above analysis, we hypothesize:

**Hypothesis 2**. Corporate strategic deviance has a significant mediating effect on the relationship between officials’ promotion expectation and corporate growth.

### 2.3 The moderating effect of corporate ownership between corporate strategic deviance and corporate growth

State-owned enterprises (SOEs) are companies with ownership of government [38], exist in both developed and emerging markets [39], account for about 20% of the total global market value [40]. At the same time, ownership is one of the important elements of institutional environment [41], there are differences in agency and efficiency between state-owned enterprises and non-state-owned enterprises [42]. Therefore, this paper explores the moderating effect of corporate ownership between corporate strategic deviance and corporate growth. We argue that compared with non-state-owned enterprises, corporate strategic deviance has less influence on state-owned enterprises’ growth.

More specifically, compared with non-state-owned enterprises, corporate strategic deviance has less influence on state-owned enterprises’ growth. On the one hand, SOEs lack effective supervision mechanism. According to agency theory, enterprises with separate ownership and management have the dual agency problem, SOEs are owned by "the whole people" but " managed by the state", they likely suffer more from the dual agency problem due to lack of supervision mechanism [39]. In contrast, non-SOEs often set up monitoring systems to collect information about what managers are doing [43, 44] and evaluate managers [45]. On the other hand, SOE managers might lack incentives to pursue efficiency [46, 47]. Managers may maximize their interests when enterprises with separate ownership and management [45]. SOE managers are more likely to be appointed by government; they are managers and government agents [48]. Their strategic capabilities may be weak [49], even if they are qualified managers, they often lack motivation to pursue efficiency due to lack of profit-sharing incentives [39]. In contrast, non-SOEs often motivate managers based on evaluations [45], they have economic incentives to pursue efficiency. Therefore, we believe that compared with non-state-owned enterprises, corporate strategic deviance has less influence on state-owned enterprises’ growth.

On this basis, we propose the following:

**Hypothesis 3**. The positive relationship between corporate strategic deviance and corporate growth will be weaker when firms are state-owned enterprises.

## 3. Methodology

### 3.1 Data

Our analysis of the relationship of officials’ promotion expectation, corporate strategic deviance and corporate growth is based on data from China Stock Market and Accounting Research Database (CSMAR) for the period of 2010 to 2018. The data of Chinese listed companies in CSMAR has been widely used in management research and organization research. In this study, we remove firms with missing information, our final data consists of 20798 observations. Considering the global financial crisis and policy changes [50], we argue that the period of 2010–2018 is considered stable of global environment, this reduces the undesirable noise to our data.

### 3.2 Variables

#### Dependent variable

Corporate growth in our study is measured by sales growth. We measure corporate growth by the percentage of sales change between the next year and the current year [51, 52].

#### Independent variables

The research subject is provincial governors, the data comes from officials resumes. There are two reasons for choosing provincial governors: On the other hand, provincial officials have power in policy making, policy implementation and resource allocation, so they could exert pressure on listed firms in their jurisdiction [21]. On the other hand, provincial governors are responsible for economic development, production safety and commerce. Governments heads at all levels (such as provincial governors) have power and responsibility for affairs in their jurisdiction due to the chief responsibility in China. Therefore, we choose provincial governor as the research subject. Provincial officials must retire at the age of 65 in China. The term of provincial governors depends on the number of congresses that they can attend before 65 [53], specifically in our research period the congresses is in 2012 and 2017 respectively. Officials’ promotion expectation measured by whether officials could participate in next congress, that is, whether they will be over the retirement age of 65 at next congress. If governor is official who ineligible to as a candidate and we coded it as 0, otherwise it is 1 [54].

#### Corporate strategic deviance

Corporate strategic deviance reflects the deviation between corporate strategy and industry mainstream strategy. Mintzberg suggests that corporate strategy is reflected in the resource allocation of important functional activities (such as marketing, innovation, and production) [55]. Based on this view, studies in accounting, finance, economics, and management often use data from financial statements to measure corporate strategy, for example, Bertrand and Schoar use R&D investment and advertising costs to measure corporate strategy [56]; Bentley et al. measure strategies (prospector, analyzer and defender) by R&D expenses, capital intensity, employee ratios, employee volatility and sales growth [57]. Similarly, scholars measure corporate strategic deviance by advertising intensity, R&D intensity, capital intensity, renewal of fixed assets, indirect cost efficiency and financial leverage based on Mintzberg’s view due to these data respectively determine the resource allocation of corporates in marketing, innovation, production, market expansion, operation and financing [58–60]. In this study, we follow their methodology to measure corporate strategic deviance. Since advertising intensity and R&D intensity are rarely disclosed separately in China, we follow exiting method to replace advertising expenses with sales expenses and replace R&D intensity with intangible assets [61]. The steps are as follows: we calculate advertising expenses, R&D intensity, capital intensity, renewal of fixed assets, indirect cost efficiency, financial leverage; then calculate the difference between each index and industry average, standardize and take the absolute value; at last, we get strategic deviance by calculate the arithmetic mean of each indicator.

#### Corporate ownership

We measure ownership by a dummy variable to indicate whether a firm was an SOE. Since the ownership structure is relatively stable, it is relatively objective to regard ownership as a dummy variable. Therefore, we code the state-owned enterprise (SOE) as 1, otherwise it is 0.

#### Control variables

We control several variables that may influence corporate growth. First, we measure equity restriction by the number of shares held by the largest shareholder / the total number of shares from second to tenth shareholder. Second, we control a firm’s asset liability by total load/total assets. Third, we measure firm size by number of employees (logged).

### 3.3 Model definition

According to the characteristics of research samples and the results of Hausman test (Prob = 0.000 which less than 0.05, as shown in Table 1), we used the entity fixed effect model to test our hypothesizes.

**Table 1:** The Hausman test results of officials’ promotion expectation and corporate growth.

	fe	re	Difference	S.E.
Equity restriction	-0.0388885	0.177206	-0.2160945	0.0380298
Asset liability	0.0769542	0.0488833	0.028071	0.013379
Firm size	-0.2532361	-0.0749616	-0.1782745	0.0141423
Officials’ promotion expectation	0.0327602	0.0204054	0.0123549	0.0052565
chi2	201.47			
Prob>chi2	0.000			

The regression models are constructed to argue that officials who have high promotion expectation have a positive effect on corporate growth; officials who have high promotion expectation have a positive and indirect effect via corporate strategic deviance on corporate growth; compared with non-state-owned enterprises, corporate strategic deviance has less influence on state-owned enterprises’ growth, as shown in Eqs 1, 2, 3, 4 and 5.


$$CG_{it+1}=\alpha_{0}+\alpha_{1}\times OPE_{it}+\alpha_{2}\times ER_{it}+\alpha_{3}\times AL_{it}+\alpha_{4}\times FS_{it}+\tau_{i}+\mu_{it}$$ (1)

$$CSD_{it+1}=\beta_{0}+\beta_{1}\times OPE_{it}+\beta_{2}\times ER_{it}+\beta_{3}\times AL_{it}+\beta_{4}\times FS_{it}+\tau_{i}+\mu_{it}$$ (2)

$$CG_{it+1}=\gamma_{0}+\gamma_{1}\times CSD_{it}+\gamma_{2}\times ER_{it}+\gamma_{3}\times AL_{it}+\gamma_{4}\times FS_{it}+\tau_{i}+\mu_{it}$$ (3)

$$CG_{it+1}=\delta_{0}+\delta_{1}\times OPE_{it}+\delta_{2}\times CSD_{it}+\delta_{3}\times ER_{it}+\delta_{4}\times AL_{it}+\delta_{5}\times FS_{it}+\tau_{i}+\mu_{it}$$ (4)

$$CG_{it+1}=\varepsilon_{0}+\varepsilon_{1}\times CSD_{it}+\varepsilon_{2}\times CS_{it}+\varepsilon_{3}\times CSD_{it}\times CS_{it}+\varepsilon_{4}\times ER_{it}+\varepsilon_{5}\times AL_{it}+\varepsilon_{6}\times FS_{it}+\tau_{i}+\mu_{it}$$ (5)

## 4. Results

Table 2 reports the descriptive statistics and the correlation coefficients of the variables used in our study.

**Table 2:** Descriptive statistics and pearson correlation.

	Mean	St. D	Equity restriction	Asset liability	Firm size	Officials’ promotion expectation	Corporate strategic deviance	Corporate ownership	Corporate growth
Equity restriction	0.2417	13.7851	1.0000						
Asset liability	0.4205	0.2749	-0.197***	1.0000					
Firm size	3.3129	0.5585	-0.102***	0.254***	1.0000				
Officials’ promotion expectation	0.9122	0.2830	-0.022***	0.012*	0.054***	1.0000			
Corporate strategic deviance	0.5549	0.3622	-0.031***	0.101***	-0.153***	0.065***	1.0000		
Corporate ownership	0.3691	0.4826	‘-0.328***	0.251***	0.294***	0.072***	-0.001	1.0000	
Corporate growth	0.1838	0.4207	0.062***	-0.005	-0.097***	0.0070	0.087***	‘-0.084***	1.0000

* p<0.1;

** p<0.05;

*** p<0.01

Table 3 presents the regression results of Hypothesis 1 and Hypothesis 2. Models 1 and 2 examine the direct effects of officials’ promotion expectation on corporate growth. The coefficients of officials’ promotion expectation are positive and significant when predicting corporate growth(β = 0.0328, t = 2.84), supporting Hypothesis 1.

**Table 3:** The regression results of officials’ promotion expectation, corporate strategic deviance and corporate growth.

Model	1	2	3	4	5	6
Variable	Corporate growth	Corporate growth	Corporate strategic deviance	Corporate strategic deviance	Corporate growth	Corporate growth
Equity restriction	-0.0446	-0.0389	-0.0738***	-0.0622**	-0.0339	-0.0299
	(-1.02)	(-0.89)	(-2.76)	(-2.33)	(-0.78)	(-0.69)
Asset liability	0.0765***	0.0770***	0.1772***	0.1780***	0.0506***	0.0513***
	(4.41)	(4.43)	(16.66)	(16.78)	(2.90)	(2.94)
Firm size	-0.2483***	-0.2532***	-0.1603***	-0.1703***	-0.2249***	-0.2287***
	(-16.52)	(-16.74)	(-17.42)	(-18.43)	(-14.89)	(-15.03)
Officials’ promotion expectation		0.0328***		0.0662***		0.0232**
		(2.84)		(9.40)		(2.02)
Corporate strategic deviance					0.1461***	0.1443***
					(11.89)	(11.72)
_cons	0.9850***	0.9699***	1.0294***	0.9989***	0.8346***	0.8257***
	(19.44)	(19.04)	(33.18)	(32.10)	(16.04)	(15.81)
N	20798	20798	20798	20798	20798	20798
R-sq	0.016	0.016	0.030	0.035	0.024	0.024
Sobel *z*	(z = 7.476, p < 0.05)					

* p<0.1;

** p<0.05;

*** p<0.01

To test Hypothesis 2, we analyze the positive and indirect effect of officials’ promotion expectation on corporate growth. Following Baron and Kenny’s procedure [62]: Step 1 is to show that a significant relation exists between officials’ promotion expectation and corporate growth; step 2 is to show that there is a significant relation between officials’ promotion expectation and corporate strategic deviance; step 3 is to show that exists a significant relation between corporate strategic deviance and corporate growth; step 4 is to show that the effect of officials’ promotion expectation on corporate growth is less when corporate strategic deviance is included in the model. We can conclude that corporate strategic deviance has a significant mediating effect on the relationship between officials’ promotion expectation and corporate growth if the four conditions are met.

Models 2,4,5 and 6 test the mediation effects of corporate strategic deviance. Results in Model 2 suggest that officials’ promotion expectation has a positive and direct effect on corporate growth(β = 0.0328, t = 2.84); results in Model 4 suggest that there is a significant and positive relation between officials’ promotion expectation and corporate strategic (β = 0.0662, t = 9.40); results in Model 5 suggest that corporate strategic deviance has a positive and significant effect on corporate growth (β = 0.1461, t = 11.89); and as reported in Model 6, corporate strategic deviance reduces the strength of the effects of officials’ promotion expectation on corporate growth (from (0.0328, t = 2.84) to (0.0232, t = 2.02)).

At the same time, we use Sobel test to test the indirect effect of officials’ promotion expectation [63], the result (z = 7.476, p < 0.05) provides significant evidence of the mediation effects of corporate strategic deviance. The results support Hypothesis 2, in that corporate strategic deviance partially mediates officials’ promotion expectation—corporate growth relationship.

In Table 4, Model 9 and Model 10 test the moderating effects of corporate ownership and implies that Hypothesis 3 is supported. The results show that the coefficient estimate of the interaction terms between corporate strategic deviance and corporate ownership is negative and statistically significant (β = −0.1239, t = −5.25), thus supports Hypothesis 3. Specifically, compared with non-state-owned enterprises, corporate strategic deviance has less influence on state-owned enterprises’ growth.

**Table 4:** The moderating effect of corporate ownership.

Model	7	8	9	10
Variable	Corporate growth	Corporate growth	Corporate growth	Corporate growth
Equity restriction	-0.0446	-0.0339	-0.0365	-0.0318
	(-1.02)	(-0.78)	(-0.84)	(-0.73)
Asset liability	0.0765***	0.0506***	0.0506***	0.0513***
	(4.41)	(2.90)	(2.90)	(2.94)
Firm size	-0.2483***	-0.2249***	-0.2247***	-0.2252***
	(-16.52)	(-14.89)	(-14.88)	(-14.92)
Corporate strategic deviance		0.1461***	0.1460***	0.2009***
		(11.89)	(11.88)	(12.45)
Corporate ownership			-0.0417	0.0445
			(-1.37)	(1.28)
Corporate strategic deviance* Corporate ownership				-0.1239***
				(-5.25)
_cons	0.9850***	0.8346***	0.8503***	0.8134***
	(19.44)	(16.04)	(15.96)	(15.15)
N	20798	20798	20798	20798
R-sq	0.016	0.024	0.024	0.025

* p<0.1;

** p<0.05;

*** p<0.01

**Fig 1:**
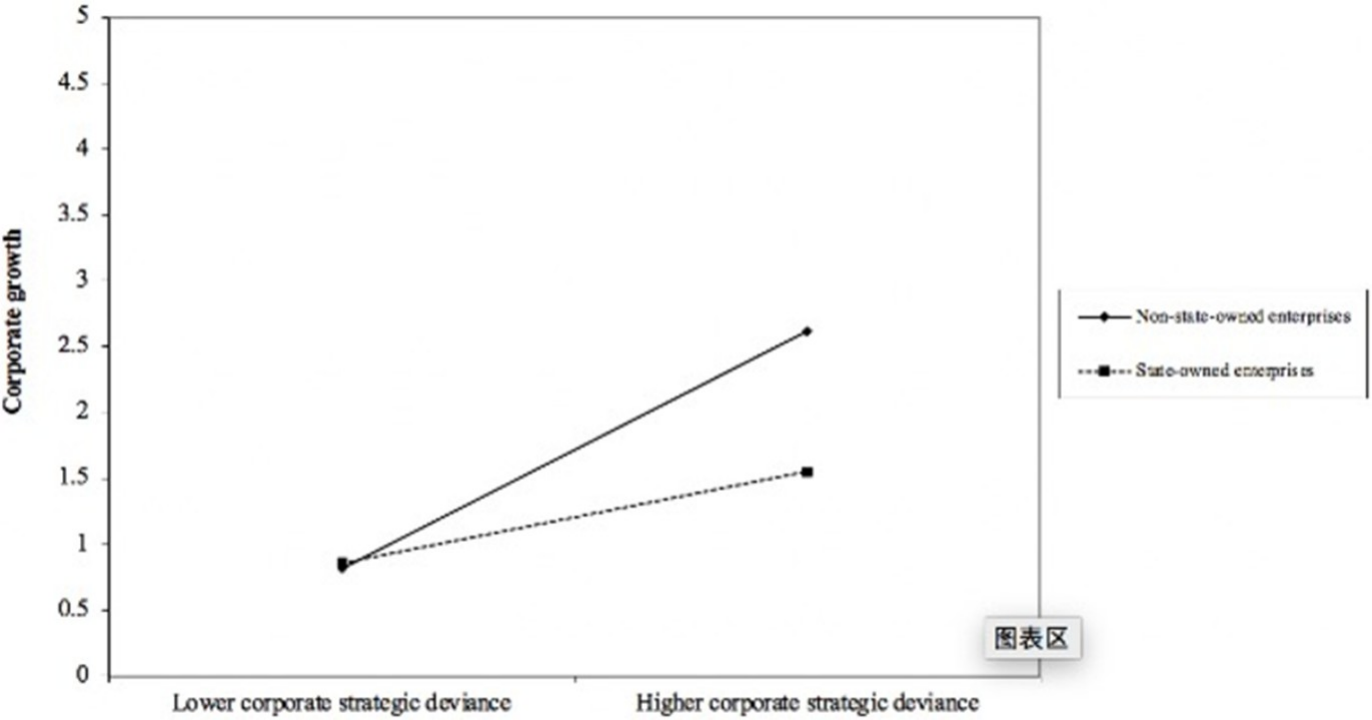
Corporate strategic deviance and corporate growth: The moderating effect of corporate ownership.

Fig 1 confirms that when enterprise is a state-owned enterprise, the positive relationship between corporate strategic deviance and corporate growth become weaker.

## 5. Discussion

Understanding whether and how government officials influence corporate growth is important not only for improving corporate competitiveness, but also for promoting national economic development. Conventional studies on the determinants of corporate growth focus on resources, strategy, and environment, for example, scholars often suggest that entrepreneurs’ characteristics, intellectual property and industrial environment are important factors influencing corporate growth [10, 11, 15, 64]. New institutional economists also suppose that institution is important to corporate growth and economic development [1, 2], but China does not have advantages in property, capital market, legal and other aspects according to the criteria of LLSV. More and more scholars believe that government (especially local government) plays an important role in China’s economic growth [3], and government is made up of officials. Although existing research have explored the influence of officials’ promotion pressure on economic growth, few research investigate the influence of officials’ promotion expectation on corporate growth.

To address the direct influence of officials’ promotion expectation on corporate growth, we examined data from Chinese listed companies between 2010 and 2018, the empirical results show that officials’ promotion expectation has a significantly positive impact on corporate growth; corporate strategic deviance has a mediating effect on the relationship between officials’ promotion expectation and corporate growth; compared with non-state-owned enterprises, corporate strategic deviance has less influence on state-owned enterprises’ growth.

The positive relationship between officials’ promotion expectation and corporate growth is reported in this study. Specifically, officials who as "rational economic person" will transfer their political goals and social tasks to corporates and promote corporate growth by intervene in enterprises activities, provide scarce resources and policy support [65]. This finding is consistent with Weber’s view that government effectiveness in modern countries depends neither on parliamentary debate nor on the state leaders’ edict, but the civil servants who carry out the day-to-day administration affairs [66].

The second finding is that there is an indirect relation between officials’ promotion expectation and corporate growth. That is, the role of officials’ promotion expectation is path dependent, corporate strategic deviance represents the intermediate channels that account for the effects of officials’ promotion expectation on corporate growth. This finding provides a useful exploration for clarifying the internal influence mechanism of government officials on corporate growth.

Another important finding of this research is the moderating effect of corporate ownership between corporate strategic deviance and corporate growth. This means that corporate ownership are the important boundary conditions for corporate strategic deviance to promote corporate growth. Although previous studies have emphasized the difference between SOEs and non- SOEs in resource acquisition [48, 67, 68], we find that the positive effect of corporate strategic deviance is inhibited due to state-owned enterprises are limited by agency problems and efficiency problems.

## 6. Implications

The research significance of this study is reflected as follows. First, this study riches the research of stakeholders and agency theory. We examine how officials’ promotion expectation promote corporate growth based on political sociology and political economy, the finding provides a political explanation for corporate growth. Second, this study introduces one pathway which officials’ promotion expectation affects corporate growth, generates a more comprehensive understanding of the relationship between political stakeholders and corporate growth. The impact of officials on corporate growth is a complex phenomenon. Demystifying it entails studying the path dependent role of officials’ promotion expectation and intermediate factors that mediate the relationship between officials’ promotion expectation and corporate growth. We extend the intermediate factors research by demonstrating one intermediate channels with corporate strategic deviance. Third, the result shows that corporate ownership are the important boundary conditions for corporate strategic deviance to promote corporate growth, this helps explain why different companies implement the same strategy but have different effects and enriches the cognition of corporate strategic deviance.

Our study also generates important managerial implications. How to effectively evaluate and motivate government officials is an important and difficult issue [6, 69]. The finding that officials’ political demands have a positive impact on corporate growth bring enlightenment to officials’ governance. The central government should establish a scientific and objective assessment mechanism. The assessment indicators need to refine along with social development and include multiple aspects (such as economic, social and ecological); the evaluation method should be quantitative, and the indicators’ weight should be set scientifically to avoid the uncertainty of subjective judgment. At the same time, the finding suggests that enterprises should fully consider the possible influence of officials when they make strategic planning and pursuit growth. “Promising government" is conducive to corporate strategic deviance and corporate growth.

In addition, our findings have implications for governments and enterprises in other countries. Although China’s system characterized by political centralization and economic decentralization has its particularity, it also has some similarities with other political systems, such as the federalism in United States. Just as Besley and Case found that the governors’ re-election prospects in American largely depended on the economic performance of their states compared with other states [70], this is similar to China. However, the findings may not apply to countries that political power is high concentrations(such as Russia and Malaysia) because the official mobility is low [71].

## 7. Limitations and conclusions

There are some limitations in this study. First, officials’ promotion expectation may be affected by personality, ability and comprehensive quality, future studies should quantify these indicators to enrich officials’ promotion expectation research. Second, corporate strategic deviance not only has an impact on corporate growth, but also on corporate profit. In future studies, we should explore the impact of corporate strategic deviance on corporate profit. Third, this study focuses on the role of officials’ promotion incentives on corporate growth but does not consider the possible impact of executive’s characteristics (such as CEO career concerns, CEO experience, CEO equity incentives). Executives with different characteristics may respond differently to officials’ demands, future studies can explore the impact of the interaction between government officials and executives on corporate growth.

In summary, this study examined how officials’ promotion expectation influence corporates—directly and indirectly via corporate strategic deviance—in terms of corporate growth. Using Chinese listed companies from 2010 to 2018 as research samples, we find that officials who have high promotion expectation have a positive effect on corporate growth; corporate strategic deviance has a significant mediating effect on the relationship between officials’ promotion expectation and corporate growth; the positive relationship between corporate strategic deviance and corporate growth become weaker when firms are state-owned enterprises. The research generates a more comprehensive understanding of the political stakeholders-corporate growth relationship.
